# Clinical and microbiological profile of infectious keratitis in children

**DOI:** 10.1186/1471-2415-13-54

**Published:** 2013-10-16

**Authors:** Patricia Chirinos-Saldaña, Victor Manuel Bautista de Lucio, Julio Cesar Hernandez-Camarena, Alejandro Navas, Arturo Ramirez-Miranda, Lizet Vizuet-Garcia, Mariana Ortiz-Casas, Nadia Lopez-Espinosa, Carolina Gaona-Juarez, Luis Antonio Bautista-Hernandez, Enrique O Graue-Hernandez

**Affiliations:** 1 Cornea and Refractive Surgery Department, Institute of Ophthalmology “Fundación de Asistencia Privada Conde de Valenciana”, Mexico City, Mexico; 2 Microbiology and Ocular Proteomics, Research Unit, Institute of Ophthalmology “Fundación de Asistencia Privada Conde de Valenciana”, Mexico City, Mexico

**Keywords:** Paediatrics, Children, Drug-resistance, Microbial, Risk factors

## Abstract

**Background:**

Infectious keratitis is a sight-threatening condition for children. The purpose of this study was to describe the clinical profile, risk factors and microbiological profile of infectious keratitis in children.

**Methods:**

Retrospective review of clinical records of patients under 16 years of age with history of microbial keratitis seen at a tertiary referral center. Clinical characteristics, risk factors, visual and surgical outcomes as well as the microbiological profile are analyzed.

**Results:**

Forty-one eyes of 41 patients. Mean age was 8.7 years. Time between the onset of symptoms and ophthalmological examination was 12.7 days. Predisposing factors were found in 78%; ocular trauma was the most common (25%). Visual acuity equal or worse than 20/200 at admission correlated positively with a poorer visual outcome, p=0.002. Positivity of cultures was 34%. Gram-positive bacteria were isolated in 78.5%; *Staphylococcus epidermidis* (28.6%) was the most common microorganism.

**Conclusions:**

Our study emphasizes the importance of a prompt diagnosis and treatment of infectious corneal ulcers in children. Trauma and contact lenses were the main predisposing factors. Gram-positive organisms were isolated in the vast majority of cases and visual outcomes are usually poor.

## Background

Worldwide, infectious corneal disease is an important cause of visual impairment and blindness, with reported annual incidence between 1.5 to 8 million [1], being more prevalent in developing countries. Although, infectious keratitis is an uncommon event in paediatric patients, amblyopia is of concern, since altered corneal transparency during infancy prevents normal neurophysiological development [2].

According to the World Health Organization (WHO), approximately 700,000 children annually develop corneal pathology that permanently affects their vision [3]. This fact is significant because the eventual blind-years are greater when compared to adults, and so its incremental cost to healthcare systems. Incidence of blindness caused by keratitis in children is 20 times higher in tropical developing countries with poor healthcare when compared to developed countries [4]. Ocular trauma, the main predisposing factor for infectious keratitis in children, is reported in 26–58.8% of cases [5].

Corneal infections in pediatric patients differ from adult disease in the risk factors, evolution, treatment compliance and complications. These differences usually result in a poorer visual prognosis [2,6,7].

The purpose of this study is to describe the clinical, microbiological and predisposing factors of infectious keratitis in pediatric patients to improve the diagnosis, treatment and visual prognosis of this unique set of patients.

## Methods

The study was approved by the ethics committee of the Institute of Ophthalmology “Conde de Valenciana”, Mexico City. This is a retrospective review of clinical records of patients younger than 16 years with diagnosis of infectious keratitis seen at the Cornea and External Disease Unit at Institute of Ophthalmology “Conde de Valenciana”, in Mexico City between January 2006 to December 2011. Microbiological data were obtained from the Department of Microbiology and Ocular Proteomics of the same institution.

The studied variables included demographic data, medical history, risk factors (history of ocular trauma, use of contact lenses, associated eye diseases, systemic diseases, previous ocular surgery), clinical presentation, initial and final visual acuity, medication use before and after diagnosis, and need for surgical therapy.

### Ophthalmic examination

All patients had a detailed clinical evaluation followed by corneal scrapings. The material obtained on scraping was subjected to standard microbiology evaluation. Initial medical treatment was based on fourth-generation fluoroquinolones monotherapy and modified in accordance with clinical response, culture and antibiotic susceptibility results.

Every patient underwent a comprehensive ophthalmic examination, including but not limited to, presenting uncorrected distance visual acuity (UDVA) and pinhole corrected distance visual acuity (CDVA), slit-lamp biomicroscopy examination, fundoscopy and intraocular pressure. With respect to corrected visual acuity, patients were classified according to the revised ICD-10 [8]. The location, depth and size of the ulcer, as well as infiltrate appearance, presence or absence of lysis, hypopyon or neovascularization were documented at each visit. The longest diameter of the ulcer at presentation was defined as the size of the ulcer. The ulcer was defined, as being central if it involved the pupillary area otherwise it was recorded as peripheral. Follow-up was done as needed.

### Microbiology workup

In all patients, corneal scrapings were obtained, smears were prepared for standard microbiologic evaluation including Gram and Giemsa stains. The sample was sowed in Columbia agar + 5% sheep, chocolate agar + PolyViteX (PVX) and Brain-Heart Infusion (BHI), those were incubated at 37°C and 5% CO_2_; and Sabouraud dextrose agar, which was incubated at 28°C and 5% CO_2_. The bacteria were identified using the Vitek 2 Compact system (bioMérieux, France) with GP-test Vitek card. The drug sensitivity was determined by the Kirby-Baüer method using the following antibiotic discs: polymyxin, oxacillin, neomycin, sulfamethoxazole, vancomycin, gentamicin, ciprofloxacin, ofloxacin, cephalothin, cephazolin and ceftazidime and according to Clinical and Laboratory Standards Institute guidelines [9]. Criteria for culture positivity were growth of the organism at the site of inoculation on two or more solid phase cultures, or growth at the site of inoculation on one solid phase media of an organism consistent with microscopy, or confluent growth on one media.

### Statistical evaluation

The statistical analysis was performed with SPSS 17.0 software (SPSS Inc, Chicago, IL, USA). Descriptive statistics were obtained to determine the frequency and proportions. One-way analysis of variance (ANOVA) and linear regression model were used to evaluate the change of visual acuity from admission to discharge.

## Results

### Patient characteristics

Between 2006 and 2011, 41 corneal samples from 41 children with infectious keratitis were identified. Twenty-one cases were male (51%), and 20 were female (49%). The mean patient age was 8.7 years ± 5.1 (range, 3 months to 15 years). The mean time from the onset of symptoms to the ophthalmological examination was 12.7 days ± 18.7 (range, 1–60 days).

Predisposing factors were identified in 78% of cases, with 2 or more factors occurring in 26%. The most common predisposing factor was ocular trauma (25%), followed by wearing contact lenses and prolonged steroid treatment (Table 1). In cases associated with ocular trauma, pencils were the most common cause (10.6%); other was associated with fireworks, cat scratch, rope, soil and toys.

**Table 1:** Predisposing factors for infectious keratitis in children

**Predisposing factors**	**No. of patients**	**%**
**Trauma**	8	25%
**Contact lenses**	5	15.6%
**Steroid treatment**	4	12.5%
**Ocular rosacea**	3	9.4%
**Previous ocular surgery**	3	9.4%
**Systemic immunodeficiency**	3	9.4%
**Congenital facial paralysis**	3	9.4%
**Congenital anomalies of anterior chamber**	2	6.3%
**Previous herpetic infection**	1	3.1%

**Figure 1:**
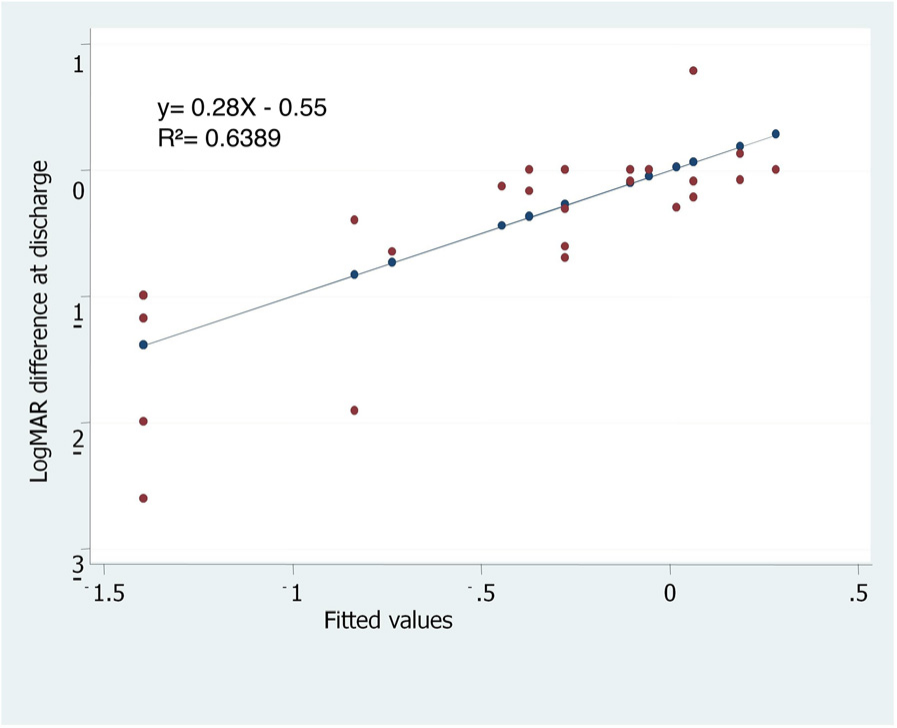
**Linear regression model: Estimation of change of visual acuity at discharge for every unit of visual acuity at admission.** Legend: An mean increment of logMAR 0.55 at discharge was observed for every increasing unit of logMAR at admission, *p* <***0***.*0001*.

Patients with CDVA ≥ 20/60 at admission showed a statistically non-significant improvement of their vision at discharge, but happened inversely when CDVA was < 20/60 (p=0.003). A worsening of visual acuity was more pronounced when CDVA was less than 20/200 at admission (mean increment of logMar 1.04, p = 0.002). Linear regression analysis showed for lower visual acuity at admission, lower visual acuity at discharge (p<0.0001) [Figure 1].

The ophthalmological examination revealed a mean epithelial defect size of 2.74 ± 1.6 mm, with visual axis involvement in 63.2% of cases, anterior chamber reaction in 31.6% and hypopyon in 15.8%.

### Treatment

As mentioned previously initial medical therapy was based on fourth generation fluoroquinolones and modified according to clinical response or antibiogram. In 26 patients (63.4%) this therapeutic regimen remained (0.5% moxifloxacin or 0.3% gatifloxacin); 6 (14.6%) were switched to macrolides (0.5% erythromycin); 5 (12.2%) to third generation cephalosporins (5% ceftazidime); 3 (7.3%) to third generation fluoroquinolones (0.3% ciprofloxacin) and 1 (2,4%) to topical 0.15% amphotericin B together with 1% natamycin and systemic oral itraconazole.

Medical therapy achieved remission in 39 cases (95%). One case developed endophthalmitis and was successfully treated with intravitreal antibiotics (vancomycin and ceftazidime). Perforation occurred in a single case and was treated with tectonic keratoplasty.

### Microbiology

Regarding the microbiological results, 66% (n=27) were negative, 26% of them (n=7) were previously treated with topical antibiotics. Cultures were positive in only 34% (n= 14), which identified 7 different microorganisms and no polymicrobial infections. Bacteria were responsible for infection in 93% (13) and fungi (*Microsporum gypseum*) in 7% (n=1).

Gram-positive bacteria were isolated in 79% (n=11) of the positive cultures. *Staphylococcus epidermidis* was the most common isolate followed by equal frequencies of *Streptococcus spp.,Corynebacterium spp.* and *Pseudomonas aeruginosa*. No significant association between risk factors and culture positivity was encountered (Table 2).

**Table 2:** Microorganisms isolated from corneal ulcers in children

**Microorganisms**		**Nº of positive cultures**	**% of positive cultures**
**Gram**-**positive bacteria**	***Staphylococcus epidermidis***	4	28.6%
	***Staphylococcus aureus***	1	7.1%
	***Streptococcus viridans***	2	14.3%
	***Streptococcus pneumoniae***	2	14.3%
	***Corynebacterium *** **sp.**	2	14.3%
**Gram**-**negative bacteria**	***Pseudomonas aeruginosa***	2	14.3%
**Fungi**	***Microsporum gypseum***	1	7.1%
**No growth**		27	66%

The antibiogram of *Staphylococcus* spp. isolates revealed that all isolates were sensitive to gentamicin, 80% (n=4) were sensitive to vancomycin and ciprofloxacin. Eighty percent (n=4) of these demonstrated resistance to sulfamethoxazole, and 75% (n=3) to cefazolin, oxacillin and polymyxin B. Eighty percent (n=4) of these revealed resistance to multiple antibiotics.

All *Streptococcus* spp. isolates were sensitive to ciprofloxacin, cefazolin, ofloxacin and ceftriaxone, while 75% (n=3) were also sensitive to sulfamethoxazole, vancomycin and gentamicin. Seventy five percent (n=3) were resistant to polymyxin B.

Both *Pseudomonas aeruginosa* isolates were sensitive to gentamicin and resistant to ciprofloxacin and ceftazidime.

## Discussion

Although uncommon, infectious keratitis in children is a condition that leads to an imminent risk of amblyopia and/or permanent visual loss and because of this the cost per case is very high [10].

Children may be poor historians and/or may not complain of ocular pain. Keratitis diagnosis and treatment may be delayed by parents, or by primary care physicians confusing keratitis with the less severe conjunctivitis, especially if the cornea is not severely affected and the infiltrate not macroscopically obvious. This may be reflected in our study where the mean time to diagnosis was almost 2 weeks. This fact may also explain the poor visual outcomes and highlights the importance to educate parents and primary care personnel in the importance of immediate referral whenever the cornea may be involved.

In our series, a history of trauma was the major predisposing factor, present in approximately one quarter of the cases (25%). This result is consistent with multiple microbial keratitis studies involving children where ocular trauma has been associated in up to two thirds of the cases. (26–58.8%) [2,11-13]. Corneal trauma disrupts the protective mechanism of the corneal epithelium, facilitating bacterial adhesion and accelerating penetration and replication of microorganisms [12,13]. Children are less careful than adults and do not understand the harm that is associated with dangerous objects. Plants, metals, plastic parts, fireworks and pencils may cause ocular trauma [2].

In our study, 15.6% of patients had a history of wearing contact lenses. Interactions between contact lens and ocular surface generated by chronic or improper contact lens wear such as overnight wear, may produce epithelial defects that predispose the wearer to bacterial adhesion [14,15]. Exposure to contaminated disinfectant solutions and biofilm formation are additional mechanisms that can cause corneal infection [16,17]. Although statistics are lacking, pediatric contact lens wear may be more common in populations where high myopia is prevalent and/or orthokeratology is popular. In our population, orthokeratology is rarely used and but its use should be cautiously advised specially in children with other risk factors for infections (pediatric rosacea, recurrent blepharitis) [6,18].

The influence of systemic diseases and malnutrition on the wound healing process should be considered as a predisposing factor for microbial keratitis in children [19,20]. A wide variety of factors such as low socioeconomic status, incomplete immunization profile and systemic diseases, including hypoxic encephalopathy, pulmonary stenosis, protein-energy malnutrition, multiple congenital anomalies and prematurity have been associated with severe microbial keratitis in children [2,12,13,21,22]. Jhanji and co-authors recently reviewed the role of immunization and malnutrition in corneal ulcers in children 5 years or younger [22]. The severity of protein-energy malnutrition was related significantly to the occurrence of bilateral infection, to an incomplete immunization scheme and poor socioeconomic status, however this study did not address the confounding between these variables. In our study, we found 3 cases with associated systemic diseases. Two children had psychomotor retardation and malnutrition, and the other had chronic cardiopulmonary disease.

Local predisposing factors for infectious keratitis were found in 16 cases (50%), which included chronic steroid use, ocular rosacea and previous ocular surgeries, congenital facial paralysis and previous herpetic infection. These factors, as well as dry eye, exposure keratopathy and eyelid abnormalities act as facilitators of corneal infection [11,13].

Regarding microbiological profile, we found that cultures were positive in 34% of cases, a value that was lower than those reported in other studies (48% to 87%) [20,21,23]. Self-prescribed antibiotic, microorganisms with slow growth on culture media, viral causes of keratitis, improper corneal sampling, and the inherent difficulty in getting corneal samples from pediatric patients may account for the low positivity rate observed in our study. The higher rates of culture positivity reported in the other studies, may also be explained by their use of general anesthesia or deep sedation for corneal scrapings in uncooperative patients [20,21,23].

As shown previously by other authors, Gram-positive microorganisms are the main etiological agents of infectious keratitis in children [2,7]. Over the years, an increased incidence of keratitis caused by coagulase-negative *Staphylococcus* has been reported, [12,13,20-23] and many studies consider coagulase-negative *Staphylococcus* as an important cause of endophthalmitis [24-26]. The antibiotic susceptibility of coagulase-negative *Staphylococcus* isolates is unpredictable, and that multi-resistance to antibiotics is common. Therefore, an antibiogram should be performed in the clinically significant ocular infections that arise from these organisms [24].

We observed a high resistance of *Staphylococcus* spp. to the antibiotics known for their action against gram-positive organisms, including sulfamethoxazole, first-generation cephalosporins and oxacillin; the latter used as a surrogate marker for methicillin-resistant organisms. A hundred percent of these cases were susceptible to gentamicin, and 80% to ciprofloxacin and vancomycin. Alternatively; *Streptococcus* spp. isolates were sensitive to the majority of antibiotics. Resistance to multiple antibiotics was seen in 80% of *Staphylococcus* spp. and in 25% of *Streptococcus* spp. isolates. Prolonged antibiotic therapy is known to promote the adaptation of organisms and development of specific cross-resistance mechanisms although knowledge of the local resistance trends in ophthalmic specimens is mandatory to provide a prompt and effective treatment [27,28].

In general, fluoroquinolones susceptibility profile was good across our series of positive cultures, making this class of antibiotic suitable for empiric treatment that may be modified according to the antibiogram results. Although in our setting resistance is uncommon, sites where it’s use is widespread in healthcare, resistance is a concern [29,30].

Finally, as previously reported [31], vancomycin is an effective anti-staphylococcal drug that is rarely associated with resistance, hence, it is important that this drug be reserved for treating infections that are resistant to other anti-staphylococcal antibiotics or in cases of severe corneal infections. In this report we encountered a *Staphylococcus epidermidis* strain that was resistant to vancomycin, which suggests the existence of some strains with complex resistance mechanisms in our environment.

The findings of this study should be interpreted cautiously. This study is limited by its small sample size and is subject to selection bias since it was performed at a tertiary referral eye care center, so the results here presented cannot be extrapolated to the general population. Nevertheless, our results strengthen the body of knowledge around infectious keratitis in children and contribute to a better understanding of microbial corneal ulcers in the pediatric patient, in the hope of improving their visual outcome.

## Conclusion

In conclusion, trauma and contact lenses were the main predisposing factors for infectious keratitis in patients 16 years or younger. The frequent involvement of the central cornea, the delay in reaching specialty care and low positivity of cultures may all account for the poor visual outcomes conveyed in our study.

## Competing interests

The authors declare that they have no competing interests.

## Authors’ contributions

PCS, VMBL, EOGH conceived, designed and drafted the manuscript. LVG, MOC, NLLE, CGJ, LABH performed the data collection. JCHC, ANP, ARM contributed to review and to the revision of the manuscript. All authors read and approved the final manuscript.

## Pre-publication history

The pre-publication history for this paper can be accessed here:

http://www.biomedcentral.com/1471-2415/13/54/prepub
